# The impact of working memory capacity on collaborative learning in elementary school students

**DOI:** 10.3389/fpsyg.2022.1027523

**Published:** 2022-12-02

**Authors:** Xuejiao Du, Cong Chen, Hongxin Lin

**Affiliations:** ^1^College of Education Science, Ludong University, Yantai, China; ^2^Department of Food and Medicine, Weihai Ocean Vocational College, Weihai, China

**Keywords:** working memory capacity, collaborative learning, elementary school students, individual learning, working memory resource depletion

## Abstract

Working memory capacity may be a critical factor that influences the effectiveness of collaborative learning; however, no studies have directly explored this effect. Using worked examples as learning tasks, Experiment 1 used a 2 (working memory capacity) × 2 (learning format) factorial design to examine the effects of collaborative learning versus individual learning of 4th-grade Chinese elementary school students with different working memory capacities. High-capacity learners displayed less working memory resource depletion and better transfer performance during collaborative learning than individual learning. In contrast, no differences were found among the low-capacity learners. Collaborative learning benefited high-capacity learners but not low-capacity learners, per our observations. To further optimize collaborative learning for low-capacity learners and expand the findings to heterogeneous collaborative learning, Experiment 2 adopted a 2 (member capacity) × 2 (group capacity) factorial design to explore the effects of member and group working memory capacity on collaborative learning in heterogeneous groups. High-capacity members displayed less working memory resource depletion and better far transfer performance in high-capacity groups compared to low-capacity groups. Simultaneously, all members had better near transfer performance in high-capacity groups compared to low-capacity groups. Both member and group working memory capacities influenced the effect of heterogeneous collaborative learning. However, low-capacity members only partially benefited from collaborative learning in high-capacity heterogeneous groups.

## Introduction

Collaborative learning refers to an instructional arrangement where students work together in a group to achieve a shared goal (Schreiber and Valle, 2013; Asterhan and Schwarz, 2016). Recently, collaborative learning has gained widespread attention owing to the increasing importance of working in teams to solve the ever more challenging problems in our contemporary life and work. However, its effectiveness on learning is still inconclusive (Kester and Paas, 2005). Some studies have indicated that collaborative learning is more effective than individual learning (Krause et al., 2009; Dong and Zhang, 2018). Some suggest that the difference between collaborative and individual learning effects is not significant in most situations (Clinton and Kohlmeyer, 2005; Retnowati et al., 2010). Others have demonstrated that collaborative learning is inferior to individual learning, especially when using worked examples as learning tasks (Kirschner et al., 2011b; Retnowati et al., 2017). Thus, the existing literature reveals that collaborative learning is not necessarily a more desirable alternative to individual learning. In the following section, we discuss the factors influencing this inconclusive result from both theoretical and empirical perspectives.

Cognitive load theory (CLT; Sweller et al., 2011) and collaborative CLT (CCLT; Kirschner et al., 2018) are the main theories explaining collaborative learning. First, CLT is grounded in our knowledge of human cognitive architecture (Sweller et al., 2011; Sweller, 2015). This architecture involves an unlimited long-term memory, which interacts with working memory that is limited in both capacity (Miller, 1956) and duration (Peterson and Peterson, 1959). This theory assumes that novel information will be processed by working memory before it is stored in the long-term memory. Cognitive load is thus defined as the load on working memory arising from the processing of novel information (Sweller et al., 2011). Notably, the cognitive load should not exceed the limits of the learners’ working memory, as the learning effect will be inhibited otherwise (Sweller, 2010). Consequently, CLT attempts to alleviate the overload imposed on learners’ insufficient working memory resources. One benefit of collaborative learning is the ability to share the load with others through the collaboration of several working memories. Thus, the relevant instructional and research focus extends beyond individual learning to collaborative learning (Jiang and Kalyuga, 2022). Collaborative groups are considered to be information processing systems that are made up of multiple (limited) working memories, which form a collective working memory (Kirschner et al., 2009a, 2011b). Consequently, there is a larger working space for a collective working memory than for single working memory (Kirschner et al., 2018). Collaborating learners will be able to invest less cognitive effort by offloading their cognitive effort across the working memories of several learners into the collective working memory. Hence, the risk of a learner becoming cognitively overloaded is relatively low (Kirschner et al., 2009b). The available working memory capacity can thus be used to deepen the processing of information and construct high-quality cognitive schemata (Kirschner et al., 2009a). By including relevant concepts, particularly the “collective working memory,” Kirschner et al. (2018) expanded CLT by proposing CCLT. Their research also confirmed that the superiority of collaborative learning stems from learners using each other’s working memory resources to form a collective working memory (Kirschner et al., 2009a, 2011a).

The abovementioned theories emphasize the importance of forming a collective working memory through collaborative learning. Based on this, it is plausible that differences in the working memory capacity of learners may impact the effectiveness of their collaborative learning. However, this issue still needs further investigation.

Previous empirical studies have examined numerous factors influencing the effectiveness of collaborative learning. These factors can be divided into three categories: task, group, and learner characteristics (Kirschner et al., 2011a, 2018; Janssen and Kirschner, 2020). In terms of task characteristics, previous studies have demonstrated the impact of task complexity (Kirschner et al., 2011a), task type (Retnowati et al., 2010, 2017), and the nature of the task (e.g., divergent vs. convergent; Kapur and Kinzer, 2007) on the effectiveness of collaborative learning. With respect to group characteristics, there is abundant evidence that group size (Laughlin et al., 2006), group composition (Wiedmann et al., 2012), and group member familiarity (Janssen et al., 2009) are vital factors that influence its effectiveness. It is worth noting that, in terms of learner characteristics, previous studies have concentrated mainly on the influence of learners’ prior knowledge on collaborative learning. Further, there is still conflicting information on whether learners with high or low prior knowledge are suitable for collaborative learning. Considerable evidence showed that collaborative learning works better than individual learning for learners with low prior knowledge, whereas for learners with high prior knowledge, there is no benefit of collaborative learning over individual learning (Zhang et al., 2016; Retnowati et al., 2018; Zambrano et al., 2019). In contrast, there exists extensive evidence that collaborative learning is a better option than individual learning only for learners with high prior knowledge, while learners with low prior knowledge do not really benefit from collaborative learning over individual learning (Nihalani et al., 2011; Liu et al., 2018). Other learner characteristics, such as working memory capacity, might have a more critical impact on the effects of collaborative learning, which remain to be explored.

Thus, there exists inconclusive evidence on the effectiveness of collaborative learning for specific learners. This theory suggests that constituting collective working memory is vital in collaborative learning, which can address the concerns for a learner’s working memory capacity being limited during individual learning (Kirschner et al., 2018). However, in terms of learner characteristics, previous studies have mainly focused on the impact of prior knowledge with inconsistent results. To date, no studies have explored the influence of working memory capacity on collaborative learning. Working memory capacity has been examined exclusively as an important learner characteristic (Oberauer et al., 2000; Wiley et al., 2014). For individual learning tasks, evidence has shown that working memory capacity influences cognitive processing and learning outcomes (Schüler et al., 2011; Redifer et al., 2016; Morra et al., 2019). Some have directly used measures of working memory capacity as part of their design to explore the role working memory capacity plays in learning (Dutke and Rinck, 2006; Sanchez and Wiley, 2009; Skuballa et al., 2012). Moreover, Li et al. (2019) also appealed for more research to pay attention to the importance of learner characteristics of working memory capacity on learning effect in their bibliometric analysis study. Thus, it seems reasonable to speculate that working memory capacity may be the vital learner characteristic that influences cognitive processing and outcomes for collaborative learning. In comparison, prior knowledge might be an indirect factor influencing collaborative learning by enlarging the size of chunking in working memory. In the case of learners with high prior knowledge, their knowledge stored in their long-term memory integrates multiple information elements into a single chunk, which is regarded as a single element by working memory (Paas and Sweller, 2011; Chen and Kalyuga, 2020). Therefore, it is crucial to explore the impact of more direct factors (i.e., working memory capacity) on cognitive processing and outcomes for collaborative learning after strictly controlling for the level of prior knowledge.

By choosing students who were complete novices to the content being learned as participants to control for prior knowledge, Experiment 1 aimed to compare the effect of collaborative learning with individual learning among learners with high or low working memory capacity (referred to as “high-capacity” or “low-capacity,” respectively). In addition to the learning outcomes measured by the transfer test performance, this experiment also measured differences in the working memory capacity of each group of learners, before and immediately after their learning, as an indicator of working memory resource depletion during cognitive processing. The enhancements to CLT posit that working memory resources can be temporarily depleted by heavy cognitive effort and be restored after a rest period (Chen et al., 2018; Leahy and Sweller, 2019; Chen and Kalyuga, 2020). The reduction in capacity, characterized by working memory resource depletion, can serve as an indicator of cognitive effort during the processing phase (Leahy and Sweller, 2019). Moreover, Chen et al. (2018) demonstrated that if the former cognitive processing depletes too many resources, the resource available to complete the immediately subsequent test task might be insufficient, and performance on this test task might decline. Thus, working memory resource depletion was measured to reveal the learners’ cognitive exertion during the learning phase and to explain learners’ performance in the immediately subsequent transfer tests under different conditions. Prior research indicated that after a short rest, cognitive resources could be restored (Tyler and Burns, 2008; Leahy and Sweller, 2019). Thus, for a period, learners’ working memory capacity is still a relatively stable individual characteristic.

Based on the preceding analysis regarding the vital role of working memory capacity in collaborative learning from theoretical and empirical perspectives, high-capacity learners will form a larger collective working memory to share the load, and deep cognitive processing will occur during collaborative learning. Thus, it was hypothesized that high-capacity learners experience less working memory resource depletion (*Hypothesis* 1a) and exhibit better performance for the near and far transfer tests (*Hypothesis* 1b) during collaborative learning versus individual learning. The case may differ for low-capacity learners. Empirical evidence has confirmed that requiring novices to learn and construct meanings from novel learning contents will increase the cognitive resource demands imposed on them (Carbonneau et al., 2020). Moreover, the learning contents used in this experiment were highly complex, and their collaborative group entirely consisted of low-capacity learners. Even if the low-capacity learners engaging in collaborative learning form a collective working memory, this collective working memory might not be able to cover the resources needed to process the learning tasks. Borrowing from another member who also has a low working memory capacity is difficult as they may get stuck in an unguided random search for answers (Zhang et al., 2016). Thus, for low-capacity learners, there may be no significant differences in their working memory resource depletion (*Hypothesis* 2a) and performance on near and far transfer tests (*Hypothesis* 2b) between these two learning formats.

However, we must note that the groups formed in Experiment 1 were homogeneous collaborative groups, comprising members with either high or low working memory capacity. As previously mentioned, homogeneous groups with low working memory capacity are entirely composed of members with low working memory capacity. It may be challenging for these groups to achieve the desired outcome through homogeneous collaborative learning because their collective working memory might still be insufficient. The case may differ for heterogeneous collaborative groups with both high-capacity and low-capacity members. Moreover, during their daily classroom practice, the working memory capacity of members in collaborative groups tends to vary considerably; in most situations, collaborative groups are usually heterogeneous. The literature also indicates that heterogeneous groups are a much more ideal alternative than homogeneous groups (Johnson and Johnson, 1994; Lou et al., 1996; Zhang et al., 2016). For example, Wiedmann et al. (2012) suggested that collaborative groups should include at least one high-ability member. Zhang et al. (2016) argued that high-prior-knowledge members in heterogeneous collaborative groups could guide and assist low-prior-knowledge members. It is worth exploring whether working memory capacity also influences the effect of heterogeneous collaborative learning. Besides, whether participation in heterogeneous collaborative groups helps low-capacity learners achieve the desired learning outcomes also remains to be determined. Notably, the groups formed in the collaborative learning format of Experiment 1 were homogeneous collaborative groups: low-capacity members formed the groups with a low capacity, and high-capacity members formed the groups with a high capacity. The capacity of each member and capacity of the group as a whole were consistent. However, the case of heterogeneous collaborative groups is somewhat different. Learners’ working memory capacity considered in the homogeneous collaborative learning format of Experiment 1can be observed from two perspectives for the heterogeneous collaborative groups in Experiment 2: the capacity of each member, referred to as “member capacity,” and the capacity of the group as a whole, referred to as “group capacity.” Consequently, the research questions were specified as follows: Do member and group capacities influence the effect of collaborative learning in heterogeneous groups? Does taking part in high-capacity groups further improve the learning effect of members (especially lower-capacity members) compared to those in low-capacity groups?

To answer these questions, Experiment 2 further compared the learning effect of heterogeneous collaborative learning with different working memory capacity members from different working memory capacity groups on their working memory resource depletion and transfer test performance. Based on CLT (Sweller et al., 2011) and CCLT (Kirschner et al., 2018), it was hypothesized that both member and group capacity influence the abovementioned learning effect of the heterogeneous collaborative groups (*Hypothesis* 3). Participating in high-capacity groups could further improve members’ learning effect compared with those in low-capacity groups. Thus, both high-capacity and low-capacity members see less working memory resource depletion (*Hypothesis* 4a) and exhibit better performance on the near and far transfer tests (*Hypothesis* 4b) in high-capacity groups than in low-capacity groups.

## Experiment 1

### Method

#### Participants

Our participants included 140 pupils, with an average level of academic attainment, from the 4th grade of an elementary school situated in a midsize city in China. None of the participants had prior knowledge of the learning content as it was not in the rigorous national curriculum before 4th grade. Their teachers further confirmed that the learning content had not been taught before. All participants were selected according to their performance in a prior knowledge test conducted to ensure that they were complete novices. Nine students who did not pass the prior knowledge test were excluded. The remaining 132 participants consisting of 55 girls and 77 boys (mean age = 9.95 years, *SD* = 0.73) were grouped by their working memory test scores. According to the scores, they were divided into two types: high and low working memory capacity, with 66 participants in each type. Then, each type of participant was randomly assigned to either collaborative learning or individual learning formats, with 33 participants in each format. An *a priori* analysis (effect size *f* = 0.25; α = 0.05, 1-*β* = 0.80) revealed that the experiment required 128 participants to reliably test the hypotheses. The sample size used in this experiment was large enough to detect medium effects. All participants gave verbal consent, and their parents signed written informed consent for their participation in the study. Ethical approval was granted by the Research Ethics Committee of Ludong University in China (no. LDU-IRB2021090105).

#### Design

This study was conducted using a 2 (working memory capacity) × 2 (learning format) between-participants design. Working memory capacity was categorized into two levels: high-capacity and low-capacity. According to the classification criteria described in Chen and Wang (2006) and the working memory test scores of the participants in the current experiment, learners with a high-capacity level had a working memory capacity of 5.5 or more, and those with a working memory capacity of 4 or less had a low-capacity level. The learning format included collaborative and individual learning. The collaborative learning format required three participants to form a collaborative group and complete the learning phase together. The individual learning format required each participant to complete the learning phase independently. Working memory resource depletion during the learning phase and performance on the near and far transfer tests were chosen as the dependent variables.

#### Materials

The word problem for a linear equation with one unknown (hereafter referred to as “equation word problem”) was chosen as the content to be learned. This equation word problem is a vital and compulsory part of the national curriculum. It poses a certain degree of difficulty for 4th-grade participants who have not yet learned it. Based on previous research (Retnowati et al., 2017) and local mathematics textbooks, the materials were designed and modified to suit the local classroom context by a teacher from the elementary school and a graduate student majoring in educational psychology (see Appendix A).

##### Prior knowledge test

A prior knowledge test was performed to select the novice participants using four problems. The first two were used to assess the learners’ basic knowledge necessary to learn the equation word problem, which involved mixed operations of addition, subtraction, multiplication, and division. Each required two steps. The last two involved solving linear equations with one unknown, which was the content to be learned and required four steps. One point was scored for each correct step. The participants chosen for the formal experiment had mastered the basic knowledge and were novices in solving simple equations. Thus, only those who solved the first two problems correctly and the last two problems incorrectly were chosen as the study participants. The reliability (Cronbach’s alpha) for the whole sample was 0.686. The relatively low reliability on the prior knowledge test might be explained by the fact that lack of prior knowledge and floor effects. Ten experts in the field were invited to separately judge the representativeness of the prior knowledge test about its measured contents. The content validity rate was 0.700.

##### Working memory test

**Figure 1:**
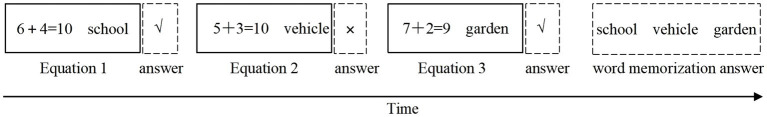
Example of a correctly solved Level 3 trial in the working memory test (translated from Chinese).

Based on Turner and Engle (1989), a working memory test for these participants was developed. The test had five difficulty levels with three trials in each, which correspond to five working memory capacity levels. The levels ranged from 2 to 6 (Level 2 contained two equation-word pairs per trial; Level 6 contained six equation-word pairs per trial). The working memory test included a memory task interrupted by a processing task. Specifically, the memory task required the participants to remember the word that followed each equation. In contrast, the processing task required them to determine whether the equation was correct or incorrect (by marking a “√” or a “×,” respectively, on the answer sheet). As shown in the example of Level 3 in Figure 1, after observing Equation 1, the participants were required to indicate whether the equation shown was correct or incorrect while keeping in mind the word that followed the equation. Then, Equation 2 and Equation 3 were done similarly. At the end of each trial, the participants were required to write down all the words in the sequence of their display. Thus, the test had a total of 60 equation-word pairs. The equations comprised arithmetic problems involving one-digit addition or subtraction; balanced addition and subtraction equations and balanced correct and incorrect equations were considered. Random words from the participants’ language textbooks were presented after the equations.

##### Learning materials

Learning from worked examples was precisely the task considered in the abovementioned studies with inconsistent results (Krause et al., 2009; Retnowati et al., 2010, 2017) and have been well proven to be effective in helping learners (especially novices) acquire novel information (Atkinson et al., 2000); hence, they were selected as the learning task in this study in an attempt to resolve the conflicting results. The learning materials contained four worked examples of equation word problems. The worked examples were designed based on their definition (Hancock-Niemic et al., 2015). Thus, each consisted of an initial problem statement, solution steps, and the final solution. The initial problem statement was an equation word problem based on real-life scenarios. For example, “There are 37 deer in the zoo; the number of deer is five times the number of tigers plus two more. How many tigers are there in the zoo?” This was followed by four steps: find the equivalence relation, construct the equation, solve the equation, and draw a conclusion (i.e., the final solution).

##### Transfer tests

Near and far transfer tests were designed to test the learning outcome. The near transfer test consisted of three problems with similar structural features but different surface features (e.g., real-life scenarios). The far transfer test consisted of three problems with different structural and surface features (e.g., a more complex equivalence relation combination of several variables). All problems required four steps. One point was scored for each correct step; the maximum score for both near and far transfer tests was therefore 12 points. The reliability (Cronbach’s alpha) for the near and far transfer tests were 0.935 and 0.886, respectively. The content validity rates were 0.800 and 0.733, respectively.

The working memory test was displayed on the participants’ 17-inch computer screens with 1,024 × 768 pixels in resolution. The prior knowledge test, answer sheet for the working memory test, learning material, and transfer tests were printed on A4-size papers.

#### Procedure

This experiment was conducted in the authentic classrooms of the abovementioned school during the first session of the school day. The general procedure included the prior knowledge test phase, working memory pretest phase, learning phase immediately followed by the working memory posttest phase, and the transfer test phase. Participants worked throughout the whole procedure under the instruction and supervision of the research assistants.

##### Prior knowledge test phase

All participants were asked to complete a prior knowledge test for 5 min on the day before the formal experiment. Only those who met the prior knowledge test requirements were chosen to participate in this experiment.

##### Working memory pretest phase

A working memory test was independently conducted on participants. The participants were required to read and complete a general instruction slide, followed by a practice task to ensure they understood the task. In a formal working memory test, each equation-word pair for each trial was shown for 6 s, then another 6 s for participants to mark a “√” or a “×” to represent the correct or incorrectness of each equation. For each trial, it was immediately after the accuracy of the last equation had been marked that participants needed to write down in sequence the memorized words that followed each equation, with 6 s provided for each word. If two or more of the three trials at each level are answered correctly (i.e., all equations are judged correctly, and the corresponding words are recalled correctly in order), the test proceeds to the higher level; otherwise, the test ends. The participants’ working memory capacity is the number of the highest level achieved. If only one trial is answered entirely correctly, the working memory capacity is the number of the previous level plus 0.5. Based on this, participants were categorized into high-and low-capacity types. They were then randomly assigned to the collaborative or individual learning formats.

##### Learning phase

During this phase, an experimental variation of the learning format was used. Collaborative learning and individual learning formats were designed based on previous studies (Dong and Zhang, 2018; Samura, 2018). In the collaborative learning format, participants were randomly allocated to collaborative groups with three participants in each group. Three participants per group were chosen because this group size was used in most previous studies and was proven optimal (Laughlin et al., 2006). Participants were also familiar with working in this group size. Before learning, the research assistants read the instructions aloud and encouraged participants to interact, elicit understanding, and help each other. They were also required to ensure that every member understood the learning material in the free discussion and discouraged non-task conversations. They were then allowed to study the worked examples individually for 5 min, followed by 10 min of free discussion within the group. In the individual learning format, participants were required to study the worked examples individually for 15 min. Moreover, any communication was not allowed.

##### Working memory posttest phase

The working memory test was administered again right after the learning phase. The difference in scores between the two working memory tests before and after the learning phase was the working memory resource depletion.

##### Transfer test phase

Participants then were required to complete the near and far transfer tests independently within 20 min. The calculations for each step of the problem should be written out. The research assistants gathered the test papers and scored their answers.

### Results

Table 1 shows descriptive statistics. Data were analyzed using a 2 (working memory capacity) × 2 (learning format) analysis of variance (ANOVA). Partial eta-squared was taken to benchmarks for effect sizes, with values of 0.01, 0.06, and 0.14 corresponding to small, medium, and large effects, respectively (Cohen 1988).

**Table 1:** Results (means and standard deviations) for the dependent variables of Experiment 1.

	High-capacity		Low-capacity	
	Collaborative learning	Individual learning	Collaborative learning	Individual learning
Working memory resource depletion	1.50 (0.91)	1.88 (0.89)	0.62 (0.81)	0.38 (0.84)
Near transfer test performance	5.91 (3.73)	2.58 (2.56)	1.73 (2.00)	0.82 (1.74)
Far transfer test performance	1.76 (3.46)	0.12 (0.33)	0.09 (0.29)	0.03 (0.17)

#### Working memory resource depletion

Concerning working memory resource depletion, the ANOVA revealed a significant main effect for working memory capacity, *F*(1, 128) = 62.54, MSE = 46.68, *p* < 0.001, *η_p_^2^* = 0.33. The main effect for learning format was not significant, *F*(1, 128) = 0.21, MSE = 0.15, *p* = 0.651. However, the interaction between main effects was significant, *F*(1, 128) = 4.27, MSE = 3.18, *p* = 0.041, *η_p_^2^* = 0.03. A simple effects test revealed that high-capacity learners experienced marginally less working memory resource depletion during collaborative learning than individual learning (*p* = 0.077). Moreover, for low-capacity learners, no significant difference was found between individual and collaborative learning (*p* = 0.257).

#### Scores from the transfer tests

Concerning scores on the near transfer test, the ANOVA revealed that the main effects of working memory capacity and learning format were both significant, *F*(1, 128) = 42.32, MSE = 291.03, *p* < 0.001, *η_p_^2^* = 0.25; *F*(1, 128) = 21.59, MSE = 148.49, *p* < 0.001, *η_p_^2^* = 0.14, respectively. The interaction between main effects was significant, *F*(1, 128) = 7.05, MSE = 48.49, *p =* 0.009, *η_p_^2^* = 0.05. A simple effects test revealed that high-capacity learners had significantly higher scores after participating in collaborative learning than individual learning (*p* < 0.001), whereas for low-capacity learners, no significant difference was found in scores on the near transfer test between individual and collaborative learning (*p* = 0.162).

Concerning scores on the far transfer test, the ANOVA revealed that the main effects for working memory capacity and for the learning format were both significant, *F*(1, 128) = 8.34, MSE = 25.49, *p* = 0.005, *η_p_^2^* = 0.06; *F*(1, 128) = 7.77, MSE = 23.76, *p* = 0.006, *η_p_^2^* = 0.06, respectively. The interaction between them was significant, *F*(1, 128) = 6.70, MSE = 20.49, *p =* 0.011, *η_p_^2^* = 0.05. Simple effects tests revealed that high-capacity learners had significantly higher scores after collaborative learning than individual learning (*p* < 0.001), whereas, for low-capacity learners, no significant difference was found in scores on the far transfer test between individual and collaborative learning (*p* = 0.888).

### Summary of Experiment 1

As shown by the results of Experiment 1, high-capacity learners experienced less working memory resource depletion and achieved better learning performance during collaborative learning, which supported *Hypotheses* 1a and 1b. In comparison, low-capacity learners saw no significant differences between collaborative learning and individual learning, even when taking working memory resource depletion and near or far transfer test performance into consideration, which was consistent with *Hypotheses* 2a and 2b. The working memory capacity affects the effectiveness of collaborative learning relative to individual learning. And the promotion of collaborative learning among low-capacity learners is far from ideal. Adding high-and low-capacity members to form heterogeneous collaborative groups may be a promising way to address this dilemma. In that case, does the working memory capacity of both groups and members affect the effectiveness of heterogeneous collaborative learning? Does taking part in high-capacity groups further improve learning effects (especially for lower-capacity members) and help lower-capacity members get the desired outcomes? Experiment 2 was conducted to answer these questions.

## Experiment 2

### Method

#### Participants

Experiment 2 recruited another 185 4th-grade students from the same elementary school who did not participate in Experiment 1. Five students who did not pass the prior knowledge test were excluded from Experiment 2. There were 180 participants consisting of 81 girls and 105 boys (mean age = 9.99 years, *SD* = 0.62) left to constitute the final sample. These participants were divided into high-and low-capacity members based on their scores on the working memory test, with 90 participants in each type. Then, each member was randomly assigned to the high-and low-capacity groups according to the number of these two types of capacity members in each group, which will be introduced in the next section. Informed consent and ethical approval were obtained from all participants.

#### Design

A 2 (member capacity) × 2 (group capacity) between-participants design was used. The member capacity included high-and low-capacity members, and the criteria for classifying high-and low-capacity members were the same as in Experiment 1. The group capacity included high-capacity groups and low-capacity groups. The former refers to the heterogeneous collaborative groups comprising two high-capacity and one low-capacity members. The latter refers to heterogeneous collaborative groups consisting of two low-capacity and one high-capacity members. The reasons for the classification of the group capacity were justified as follows: all groups were randomly assigned and worked in the same situation where collaborating to pool cognitive resources was encouraged and necessary. Moreover, the sum of three members’ working memory capacities for the high-capacity groups (*M* = 15.35, *SD* = 0.54) was significantly higher than that for the low-capacity groups (*M* = 11.95, *SD* = 0.83), *t*(58) = 18.71, *p* < 0.001, *d =* 4.86. The working memory resource depletion, near and far transfer tests performance were chosen as dependent variables.

#### Materials

All the materials in Experiment 2 were the same as those adopted in Experiment 1. In this experiment, the reliability (Cronbach’s alpha) were 0.675 on the prior knowledge test, 0.806 on the near transfer test, and 0.855 on the far transfer test.

#### Procedures

Procedures for Experiment 2 were the same as the collaborative learning format in Experiment 1.

### Results

Table 2 shows descriptive statistics. Data were analyzed using a 2 (member capacity) × 2 (group capacity) ANOVA.

**Table 2:** Results (means and standard deviations) for the dependent variables of Experiment 2.

	High-capacity members		Low-capacity members	
	High-capacity groups	Low-capacity group	High-capacity groups	Low-capacity groups
Working memory resource depletion	0.96 (0.82)	1.85 (0.90)	0.30 (0.71)	0.54 (0.88)
Near transfer test performance	3.83 (2.27)	2.23 (2.06)	2.73 (1.62)	1.10 (1.26)
Far transfer test performance	0.68 (1.63)	0.03 (0.18)	0.03 (0.18)	0.02 (0.13)

#### Working memory resource depletion

Regarding working memory resource depletion, the ANOVA showed a significant main effect for member capacity, *F*(1, 176) = 54.82, MSE = 38.68, *p* < 0.001, *η_p_^2^* = 0.24. The main effect for group capacity was significant, *F*(1, 176) = 18.21, MSE = 12.84, *p* < 0.001, *η_p_^2^* = 0.09. The interaction between them was also significant, *F*(1, 176) = 5.99, MSE = 4.23, *p* = 0.015, *η_p_^2^* = 0.03. Simple effects tests revealed that high-capacity members experienced less working memory resource depletion in high-capacity groups than in low-capacity groups (*p* < 0.001). In contrast, for high-capacity members, no significant difference was observed in the working memory resource depletion between the high-and low-capacity groups (*p* = 0.200).

#### Scores from the transfer tests

For scores on the near transfer test, the ANOVA revealed a significant main effect for member capacity, *F*(1, 176) = 14.71, MSE = 49.88, *p* < 0.001, *η_p_^2^* = 0.08, where all high-capacity members had significantly higher scores than low-capacity members. The main effect for group capacity also was also significant, *F*(1, 176) = 30.82, MSE = 104.54, *p* < 0.001, *η_p_^2^* = 0.15. Both high-and low-capacity members in the high-capacity groups had significantly higher scores than members in the low-capacity groups. However, the interaction between them was not significant, *F*(1, 176) = 0.003, MSE = 0.01, *p* = 0.954. The results suggest that both independent variables affect their near transfer test performance.

Regarding scores on the far transfer test, the ANOVA revealed that the main effects for member capacity and group capacity were both significant, *F*(1, 176) = 4.89, MSE = 4.44, *p* = 0.028, *η_p_^2^* = 0.03; *F*(1, 176) = 4.89, MSE = 4.44, *p* = 0.028, *η_p_^2^* = 0.03, correspondingly. The interaction between them was significant, *F*(1, 176) = 4.42, MSE = 4.01, *p* = 0.037, *η_p_^2^* = 0.02. Simple effects tests revealed that high-capacity members had significantly higher scores in the high-capacity groups than in low-capacity groups (*p* = 0.003). In contrast, for low-capacity members, no significant difference was observed in scores on the far transfer test between the high-and low-capacity groups (*p* = 0.938).

### Summary of Experiment 2

In conclusion, these findings demonstrated that both member and group working memory capacities influence heterogeneous collaborative learning, which supports *Hypothesis* 3. More specifically, high-capacity members experienced less working memory resource depletion and achieved better near and far transfer tests performance when in high-capacity groups compared to low-capacity groups. That is, high-capacity members profited more from participating in collaborative learning in high-capacity heterogeneous groups, which partially verified *Hypotheses* 4a and 4b for high-capacity members. Only in near transfer test performance did low-capacity members learn better in the high-capacity group than in the low-capacity group. However, no significant differences between the two groups were found when considering working memory resource depletion and far transfer test performance. Further, the results did not entirely support *Hypotheses* 4a and 4b for low-capacity members. The results suggested that low-capacity members only partially benefit from collaborative learning in a high-capacity heterogeneous collaborative group.

## Discussion

### Impact of working memory capacity on homogeneous collaborative learning compared with individual learning

Regarding working memory resource depletion, high-capacity learners experienced less depletion during collaborative learning than individual learning. In contrast, for low-capacity learners, there was no difference between collaborative and individual learning. This may be because worked examples of equation word problems used in the learning phase were complex and at a high level of element interactivity. Participants without prior knowledge must simultaneously consider multiple interactive elements during cognitive processing (Retnowati et al., 2017). For example, several variables must be simultaneously considered when constructing this kind of equation. When solving the equation, all the variables that constitute this equation must be simultaneously processed. Any changes may affect the whole equation (Chen et al., 2018). Compared to individual learning, during collaborative learning, high-capacity learners can form a collective working memory with a larger capacity and stronger information-processing potential (Kirschner et al., 2009a). The cognitive load imposed by processing this high-element-interactivity learning content can be distributed within this collective working space. This led to a considerable reduction in the cognitive effort invested by each learner; thus, the risk of the learners becoming cognitively overloaded was also lower (Kirschner et al., 2009b). Consequently, they experienced less working memory resource depletion during collaborative learning. Conversely, for low-capacity learners, no difference is observed between collaborative and individual learning in working memory resource depletion. One possible explanation may be that even though low-capacity learners engaged in collaborative learning also constructed the collective working memory, their collective working space may not be large enough to have the sufficient capacity to process and comprehend the learning content, which may cause them to experience mental exhaustion and learning burnout (Tang et al., 2021). Thus, low-capacity learners in both collaborative and individual learning formats invested less in working memory resources, and no differences in depletion were observed.

High-capacity learners showed better near and far transfer tests performance after collaborative learning than individual learning. In contrast, it did not improve for low-capacity learners. This suggests collaborative learning may be more appropriate for high-capacity learners than low-capacity learners. These results partly address the conflicting evidence regarding the effectiveness of collaborative learning (Kester and Paas, 2005; Retnowati et al., 2010; Dong and Zhang, 2018) and indicate that its effectiveness might be influenced by particular learner characteristics (i.e., working memory capacity). The following reasons may explain why collaborative learning promotes learning in high-capacity learners. First, high-capacity learners engaging in collaborative learning tend to pool their cognitive resources with other learners who also have high-capacity working memories. This allows them to construct a larger collective working memory to share the load (Kirschner et al., 2009a, 2011a). Therefore, they have more resources to devote to constructing or automating higher-quality cognitive schemata. Moreover, the effort required from each student is lowered, which leaves extra room for deeper cognitive processing of the vital content (Janssen and Kirschner, 2020). Consequently, they are more likely to engage in transactional activities for deep cognitive processing, such as negotiating the rules, justifying structural features, and providing mutual support (e.g., Van Boxtel et al., 2000; Kirschner et al., 2009b). These activities, in turn, improve learners’ motivation and interests and help them pursue ideas in depth (Qureshi et al., 2021). This ultimately enhances transfer test performance, especially far transfer test performance, which requires deep cognitive processing. Finally, as mentioned above, high-capacity learners engaging in collaborative learning depleted a lower level of working memory resources during the learning phase, leaving them with sufficient resources to perform the subsequent transfer tests. Thus, better performance is obtained.

There are several potential reasons for the lack of differences in transfer test performance between individual and collaborative learning among low-capacity learners. One observation is that the collective working memory constructed by the groups composed of low-capacity learners may be unable to provide adequate cognitive resources for processing the abovementioned learning content with a high level of element interactivity. Thus, these learners failed to construct an appropriate schema. Another possibility is that even in the collaborative learning format, low-capacity learners might still become stuck in unguided random searches during the learning phase (Zhang et al., 2016). Thus, they might only engage in superficial cognitive processing, making it challenging to understand this high-element-interactivity learning content (Kirschner et al., 2008). Third, low-capacity learners are more inclined to rely on receiving guidance from others during collaborative learning. Social loafing, where learners rely on others to perform their work, is more likely to occur in this case (Latané et al., 1979). While others with a similar low capacity in their homogeneous collaborative groups have difficulty providing such guidance, borrowing from them is also impossible (Retnowati et al., 2018). Consequently, these low-capacity learners are more likely to have negative attitudes and a depressed mental state toward their learning tasks; thus, learning burnout might occur (Tang et al., 2021). Given that they have trouble communicating the content related to learning, communication that was unrelated to learning among these low-capacity learners increased accordingly. These irrelevant transactional activities further negatively affect the learners’ and their group members’ learning (Janssen and Kirschner, 2020). Consequently, in this study, collaborative learning had no advantage in improving the learning effect of low-capacity learners.

### Effect of member and group working memory capacity on heterogeneous collaborative learning

Regarding working memory resource depletion, high-capacity members saw less depletion in the high-capacity group than in the low-capacity group. This may be because when they are in a high-capacity group where there are more high-capacity members, they do not need to provide superficial and repetitive guidance to a large number of low-capacity members. Therefore, there is relatively less working memory resource depletion. Conversely, this is what is occurring when they are in low-capacity groups with more low-capacity members (Latané et al., 1979; Webb et al., 1998); thus, the transaction costs are higher. Therefore, working memory resource depletion increases correspondingly. However, for low-capacity members, no difference was found between the two groups. The lack of difference might be explained by the possibility that all low-capacity members, whether in high-or low-capacity groups, are prone to relying on high-capacity members when they engage in collaborative learning. That is, free-riding and social loafing may be more likely to occur (Latané et al., 1979; Rajaram and Pereira-Pasarin, 2010). Moreover, the low-capacity members in both groups have few cognitive resources on their own. Consequently, they do not have as many cognitive resources to invest and exert during learning. Therefore, working memory resource depletion for low-capacity members in these two groups was low, and no difference was observed.

When considering near transfer test performance, both member and group working memory capacity influenced the outcome of heterogeneous collaborative learning. On the one hand, member working memory capacity influenced near transfer test performance in heterogeneous collaborative learning, which is consistent with studies suggesting that learners’ working memory capacity influences the learning effect (Redifer et al., 2016; Morra et al., 2019) and expands this perspective from individual learning tasks to heterogeneous collaborative learning. High-capacity members have more working memory resources to perform cognitive processing and employ more effective strategies than low-capacity members (Redifer et al., 2016). Thus, they performed better when solving near-transfer problems, whether in the high-or low-capacity groups. On the other hand, the group working memory capacity also influenced the outcome of heterogeneous collaborative learning, such that high-capacity groups formed a larger collective working memory with more resources to construct higher-quality cognitive schemata than low-capacity groups (Kirschner et al., 2009a). In line with the concept of collective working memory (Kirschner et al., 2011a), this larger collective working space also allowed members in these groups to construct more refined mental representations from this rather complex learning content. As a result, both high-and low-capacity members showed significantly better near transfer test performance in high-capacity groups than in low-capacity groups, which supported the arguments in CCLT (Kirschner et al., 2018). What is particularly noteworthy is that for low-capacity members, participation in high-capacity groups can improve their near-transfer effect compared with participation in low-capacity groups. Low-capacity members partially benefited from collaborative learning in the high-capacity group. This result might provide an alternative approach to address the undesired learning effect for low-capacity members during collaborative learning.

Regarding performance on the far transfer test, high-capacity members performed better in the high-capacity group than in the low-capacity group. The reason for this result might be that when they are in low-capacity groups, each high-capacity member needs to devote a large number of cognitive resources to guide the two low-capacity members. These explanations are primarily based on surface issues; thus, they have higher transaction costs than their peers in the high-capacity group (Janssen et al., 2010). It was difficult for them to reconcile their cognitive processing with externally provided guidance, which would be deleterious to their fine-grained schema construction and deep cognitive processing (Kalyuga et al., 2003). Thus, their far transfer test performance, which requires deep cognitive processing, was impaired. Moreover, because the high-capacity members in the low-capacity group depleted a large number of working memory resources in the learning phase, they may have insufficient resources to cover the following far transfer test, which is more difficult (Leahy and Sweller, 2019). Contrarily, high-capacity members in high-capacity groups could devote their freed cognitive capacity to do activities promoting learning and complete the test more successively. Peer pressure from other high-capacity member in their group might stimulate them to study more actively and perform tasks more efficiently (Männistö et al., 2020). Thus, high-capacity members benefited more from collaborative learning in high-capacity groups. What is particularly noteworthy is that low-capacity members did not gain significant improvement in far transfer test performance when participating in the high-capacity group; this improvement may only be reflected in the aforementioned near transfer test performance. This may be explained by the relatively high difficulty of the far transfer test. Thus, they may not have sufficient working memory resources for deeper cognitive processing (Kirschner et al., 2018). Moreover, Qureshi et al. (2021) suggested that engagement is critical for deep learning. Active engagement is a positive force for collaborative learning (Liu et al., 2022). Low-capacity members are prone to rely on high-capacity members to provide explanations; however, listening to these explanations involves only a low level of engagement. Therefore, even with participation in the high-capacity group, the performance of low-capacity members on the far transfer test did not improve. Further investigation is required to improve the deep cognitive processing of low-capacity members.

### Educational implications

This study sheds light on the relationship between working memory capacity and the effectiveness of collaborative learning, expanding our understanding of how collaborative groups are arranged and identifying an indicator of working memory resource depletion to provide a new perspective on the learning process. Based on our findings, the below pedagogical recommendations are put forward. First, teachers should organize and encourage elementary school students to learn worked example materials using a collaborative approach. However, the students’ working memory capacity must be measured and considered before learning, and the collaborative learning groups composed entirely of low-capacity students should be avoided. Second, when arranging heterogeneous collaborative groups, the working memory capacity of both the whole group and each member should be considered. Groups should be formed with the prerequisite of ensuring that the cognitive resources needed to process learning materials do not exceed the collective working memory resources of the group. For low-capacity students, arranging them in high-capacity heterogeneous collaborative groups, to a certain extent, is an alternative approach to improve their learning effect. Third, the arrangement of collaborative learning and the complexity of the learning material should be deliberately designed to ensure that working memory resource depletion is maintained within a particular scope, considering that excessive working memory resource depletion deteriorates following task performance.

### Limitations and future recommendations

First, the promotion of collaborative learning for low-capacity learners in this study was not ideal. Future studies can improve the collaborative learning effect for low-capacity learners through further working memory capacity training (or details, Shipstead et al., 2012) or support tools in computer-supported collaborative learning environments (for details, Roschelle, 2020). Second, owing to the experimental design requirements, there was considerable variation in the sample size for each condition in Experiment 2. However, we completed the verification by randomly removing some participants to ensure that each condition had the same sample size, and a consistent result was obtained. Future studies could address this issue by using different experiment designs. Third, group capacity was not measured directly. An accurate method to measure group capacity needs to be developed and adopted in future studies. Lastly, the findings suggest that excessive working memory resource depletion during the learning phase inhibits the test performance of collaborative learning. The extent to which working memory resource depletion directly influences learners’ test performance and the relationship between these two variables needs further investigation.

## Conclusion

In conclusion, learners with high working memory capacity benefit more from collaborative learning than individual learning. However, the benefit of collaborative learning over individual learning was not observed for learners with low working memory capacity. In heterogeneous collaborative learning, both member and group working memory capacities influence its effectiveness. Members with high working memory capacity benefit more from collaborative learning in high-capacity groups than in low-capacity groups. In contrast, members with low working memory capacity only partially benefit from collaborative learning in the high-capacity groups.

## Data availability statement

The original contributions presented in the study are included in the article/supplementary material, further inquiries can be directed to the corresponding author/s.

## Ethics statement

The studies involving human participants were reviewed and approved by Research Ethics Committee of Ludong University in China. Written informed consent to participate in this study was provided by the participants' legal guardian/next of kin.

## Author contributions

XD contributed to the writing and editing of the manuscript. CC processed the data and performed the data analysis. HL contributed to the design of experiments. All authors revised and approved the submitted version of the manuscript.

## Funding

The study was funded by a grant from a youth project of Shandong Province Education and Teaching Research, “Constraint Mechanisms and Optimizing Strategies of Working Memory Capacity on Collaborative Worked Examples Learning for Elementary School Students” (no: 2021JX004).

## Conflict of interest

The authors declare that the research was conducted in the absence of any commercial or financial relationships that could be construed as a potential conflict of interest.

## Publisher’s note

All claims expressed in this article are solely those of the authors and do not necessarily represent those of their affiliated organizations, or those of the publisher, the editors and the reviewers. Any product that may be evaluated in this article, or claim that may be made by its manufacturer, is not guaranteed or endorsed by the publisher.
